# Age-Related Brain Activation Changes during Rule Repetition in Word-Matching

**DOI:** 10.3389/fnhum.2017.00543

**Published:** 2017-11-13

**Authors:** Ikram Methqal, Basile Pinsard, Mahnoush Amiri, Maximiliano A. Wilson, Oury Monchi, Jean-Sebastien Provost, Yves Joanette

**Affiliations:** ^1^Centre de Recherche de l'Institut Universitaire de Gériatrie de Montréal, Montreal, QC, Canada; ^2^Faculty of Medicine, University of Montreal, Montreal, QC, Canada; ^3^Centre de Recherche CERVO - CIUSSS de la Capitale-Nationale et Département de Réadaptation, Université Laval, Québec, QC, Canada; ^4^Department of Clinical Neurosciences, Hotchkiss Brain Institute, Cumming School of Medicine, University of Calgary, Calgary, AB, Canada

**Keywords:** word-matching, rule repetition, semantic control demands, neuro-functional reorganization, healthy aging, fMRI

## Abstract

**Objective:** The purpose of this study was to explore the age-related brain activation changes during a word-matching semantic-category-based task, which required either repeating or changing a semantic rule to be applied. In order to do so, a word-semantic rule-based task was adapted from the Wisconsin Sorting Card Test, involving the repeated feedback-driven selection of given pairs of words based on semantic category-based criteria.

**Method:** Forty healthy adults (20 younger and 20 older) performed a word-matching task while undergoing a fMRI scan in which they were required to pair a target word with another word from a group of three words. The required pairing is based on three word-pair semantic rules which correspond to different levels of semantic control demands: functional relatedness, moderately typical-relatedness (which were considered as low control demands), and atypical-relatedness (high control demands). The sorting period consisted of a continuous execution of the same sorting rule and an inferred trial-by-trial feedback was given.

**Results:** Behavioral performance revealed increases in response times and decreases of correct responses according to the level of semantic control demands (functional vs. typical vs. atypical) for both age groups (younger and older) reflecting graded differences in the repetition of the application of a given semantic rule. Neuroimaging findings of significant brain activation showed two main results: (1) Greater task-related activation changes for the repetition of the application of atypical rules relative to typical and functional rules, and (2) Changes (older > younger) in the inferior prefrontal regions for functional rules and more extensive and bilateral activations for typical and atypical rules. Regarding the inter-semantic rules comparison, only task-related activation differences were observed for functional > typical (e.g., inferior parietal and temporal regions bilaterally) and atypical > typical (e.g., prefrontal, inferior parietal, posterior temporal, and subcortical regions).

**Conclusion:** These results suggest that healthy cognitive aging relies on the adaptive changes of inferior prefrontal resources involved in the repetitive execution of semantic rules, thus reflecting graded differences in support of task demands.

## Introduction

In line with the comprehensive cognitive aging model developed by Craik and Bialystok (2006), the exploration of interactions between world knowledge and the executive control processes engaged in acting upon the world, do contribute to a better understanding of age-related cognitive and adaptive changes. In healthy aging, some cognitive domains decline with age, while many others, such as language abilities, remain well-maintained throughout the lifespan (Park et al., 2002; Verhaegen and Poncelet, 2013). Specifically, older adults perform at least as similar as young adults in language comprehension and semantic processing, thus suggesting that semantic representation remains intact as we age (Pennequin et al., 2006; Maintenant et al., 2011). Moreover, there is consensus that aging has low impact on the organization of semantic knowledge as revealed by word associations and taxonomical categories (Wingfield and Stine-Morrow, 2000; Burke and Shafto, 2008). This organization is thought to be economical, being at the core of semantic knowledge acquisition and experience accumulation (Hedden and Gabrieli, 2004; Maintenant et al., 2011). Although many semantic aspects of language comprehension are spared, comprehension tasks that place high control process demands, mediated by prefrontal regions, might be more susceptible to age-related functional changes (Madden et al., 2012; Mudar et al., 2015; Diaz et al., 2016). However, investigation of age-related neurofunctional changes relevant to semantic control processing demands is scarce, as semantic processing is thought to be better preserved among the different components of language (Mayr and Kliegl, 2000; Federmeier and Kutas, 2005). More specifically, it is not clearly understood whether older adults show similar or different neural patterns to younger adults when faced with cognitive challenges in semantic tasks.

It is now well-established that cognitive abilities mediated by frontal regions are associated with dynamic/adaptive age-related neurofunctional changes even when performance is roughly similar for older and younger adults. Such neurofunctional reorganization patterns were captured in the *Hemispheric Asymmetry Reduction in OLDer adults* (HAROLD) hypothesis (Cabeza et al., 2002), as well as by the reported shift in activation from the occipito-temporal to frontal regions, known as the *Posterior-Anterior Shift in Aging* (PASA; Davis et al., 2008). In offering another comprehensive framework of age-related activation changes, the compensation-related utilization of neural circuits hypothesis model (CRUNCH, Reuter-Lorenz and Cappell, 2008) has been put forward to account for the task demands. According to this model, older adults show greater reliance on the inferior prefrontal regions at low levels of task demands, but as cognitive demands increase, limited neural resources are not sufficiently available to face a more complex cognitive challenge, resulting in a behavioral performance decline. Globally, these age-related neurofunctional changes can be conceived, at least partly, as the neurofunctional reorganization that allows the brain to sustain cognitive performance in older adults facing neurofunctional limitations. Alternatively, such neurofunctional changes could also represent the natural evolution of the neural bases of a cognitive system that enriches itself and evolves with age.

In language comprehension, it has been shown that to face an effortful retrieval and maintain a high level of performance, older adults show a greater inferior prefrontal activation than younger adults (Nielson et al., 2006; Wierenga et al., 2008; Martins et al., 2014). This suggests that neural changes underlying semantic control processes for tasks requiring cognitive demands upon these processes are taking place even as performance is maintained in older adults. Nevertheless, it remains valuable to explore if age-related brain activation changes can be observed during semantic categorization tasks, which exert much demand on effortful semantic processing.

Numerous neuroimaging studies have argued for a central role of the prefrontal region in cognitive control (Badre et al., 2005; Nagel et al., 2008; Spreng et al., 2010; Whitney et al., 2012). It is also thought to be a component of the semantic neural network underlying performance for semantic retrieval, or semantic working memory processes (Thompson-Schill et al., 1997; Wagner et al., 2001; Noonan et al., 2010). Consistent with this notion, repeated or continuous access to semantic-based knowledge appears to imply both semantic and cognitive control networks. In a meta-analysis of neuroimaging studies, Noonan et al. (2013) reported that executive-semantic processing in various language tasks modulates activations in bilateral brain networks, including the dorsolateral prefrontal cortex (PFC), ventrolateral PFC, inferior parietal cortex, and posterior temporal cortex. Similarly, Hyafil and Koechlin (2016) found that the lateral prefrontal cortex contributes to executive control processes enabling a previously running task to be maintained for subsequent retrieval. They also provided evidence that prefrontal regions are relevant for executive-semantic processing when the rules retrieved from memory do not provide sufficient information to allow for a simple or easy execution of a task. This active rule maintenance represents a critical component of the cognitive control required for successful performance in semantic categorization tasks requiring for repetitive execution of a rule (Wagner et al., 2001; Noppeney et al., 2004; Noonan et al., 2010).

A current issue in cognitive neuroscience is the study of language processing and short-term memory (STM), for the maintenance of verbal information, as dependent cognitive systems, even if the properties of each system are unique. In that regard, Majerus (2013) put forward an integrative framework germane to the short-term maintenance of verbal information and the notion of repetition in language. Given the demands of rule-based categorization, category learning necessitates the ability to actively maintain a rule. At the same time, this rule requires language repetition in STM. Thus, effective rule use depends on demand from the rule itself placed on cognitive resources. Hence, increased rule complexity will exert a greater demand on verbal STM leaving insufficient cognitive resources available to successfully perform the task. This short-term maintenance in verbal tasks involves the temporary activation of semantic representation.

Another dimension to be considered in the repetition/application of a semantic rule is the complexity of the semantic relationship between the words. In healthy younger adults, for example, Lei et al. (2010), the use of an original deductive-category reasoning paradigm showed that the detection of an item's category membership is associated with different processing times according to whether the semantic relationship between the words is typical (parrot-sparrow), or of an atypical relationship (parrot-ostrich). This result suggests that a stored representation of a given category requires greater cognitive control processes if it is less associated with the concept of the target category. Similarly, a recent study by López Zunini et al. (2014) reported that semantic decision is less effortful for words that share higher number of semantic features, or for those that are highly associated. Hence, relevant semantic information may be recruited in a top-down manner in semantic network because associated words could also be activated within neural semantic network. Interestingly, another fMRI-based finding (Jackson et al., 2015) proposes that the difference between categorical and associative relationships is more related to the level of control demand rather than the type of semantic relationship. Indeed, in categorical conditions by which the probe and target share many features, thus increasing executive demands, greater frontal activation was found as compared to associative condition. The results of this study also imply that associative and categorical relations of conceptual knowledge represent a unique and valuable dimension for manipulating task demands in language comprehension studies. These two types of semantic relationships appear to be associated with distinct levels of semantic control demands since it is known that there are also differences in executive-control processing of semantic associative and categorical relationships (Khateb et al., 2003; Kalénine et al., 2012; Mirman et al., 2017). Hence, semantic categorization represents a highly accurate and sensitive paradigm of the executive-semantic demands, which underlie cognitive processes, these being related to the nature of the semantic relationship. Taken together, these findings stress the importance of studying age-related neurofunctional reorganization during the repetition/application of semantic categorization rules.

The aim of this study was to describe age-related reorganization of the neurofunctional networks supporting semantic control demands during a word matching-task. A graded semantic strength of the functional/categorical semantic relationship between the words of a task was thought to allow a description of the neural and cognitive processes involved in the ability to repeat the application of a semantic rule, thus maintaining the continuous execution of given semantic relationships. More specifically, this study made use of a task asking for the application of either a new or already used (repeated) semantic categorisation rule in the context of a word-matching task. A word-semantic adaptation of the Wisconsin Card Sorting Test (WCST) by Monchi et al. (2001), Simard et al. (2011), and Martins et al. (2014) asked either for the repeated use of a given semantic rule, after feedback indicating the maintenance of a categorical or functional relationship, or the use of another semantic rule. Three semantic rules based on categorical vs. functional relationships were used: (a) typically related words (co-hyponyms) (e.g., dove-parakeet); (b) atypically related words (co-hyponyms) (e.g., dove-albatross); and (c) functionally related words (e.g., dove-symbol). At the behavioral level, slower response times and less accurate responses were expected under higher (low-typical) semantic processing demands than under moderate (high-typical) and relatively low semantic processing demands (functional relatedness). Two main results were expected in line with the study's aim. First, and based on the literature reported, that the repetition of the application of a semantic rule based on an atypical co-hyponymic relationship (*atypical rule*) would be processed slower and less accurately than that of a typical (*typical rule)* or a functional (*functional rule*) semantic relationship. Under such conditions, larger clusters of brain activations would be observed in higher semantic control demand when an atypical rule is applied rather than typical and functional rules, regardless of the age group. With consideration to the CRUNCH model, the second expected result was that older adults would show a longer response time and less accuracy than younger participants for the atypical rule as compared to the typical and functional rules. From a neuroimaging perspective, we expect in older adults that they would exhibit larger brain activations in the prefrontal regions when faced with increased semantic processing demands (atypical rule). Also, and independently of age, it was expected that there would be semantic task-related differences in activation within the semantic control networks.

## Materials and methods

### Participants

Twenty healthy older adults aged between 63 and 80 and 20 younger adults whose ages ranged from 19 to 35 were recruited from a pool of volunteers at the Center de Recherche de l'Institut Universitaire de Gériatrie de Montréal (CRIUGM). All participants were native French speakers and all were right-handed (scores greater than +95) as assessed by the Edinburgh Handedness Inventory (Oldfield, 1971). All had normal or corrected-to-normal vision; none had any history of major neurological disease, psychiatric illness, head injury, stroke, substance abuse, learning disabilities, or any problems that could interfere with behavior testing. Prior to the neuro-imaging session, all participants were also given a battery of neuro-psychological tests during a single 90-min session which included: screening of global cognitive function (The Montreal Cognitive Assessment, MoCA; Nasreddine et al., 2005); the inhibition measure (Stroop Test; Stroop, 1935); the flexibility measure (Trail Making Test, TMT A and B; Reitan, 1955); working memory measure (forward and backward Digit Span, WAIS III; Wechsler, 1981); several measures of ability to select a rule, maintain it, and switch to a new rule are from Burgess and Shallice (1997), for the Brixton test and Nelson (1976), for the WCST; and semantic fluency as represented by the total number of words produced in 2 min for the category Animals (Cardebat et al., 1990). Table 1 provides a detailed description of the raw cognitive measures as well as a statistical comparison of group means. Furthermore, the younger and the older adults' cognitive scores (not shown in Table 1) were within the average range according to all psychometric standardized data, suggesting normal cognitive functioning within the two groups. All participants gave written informed consent to the protocol, which was approved by the Institut Universitaire de Gériatrie de Montréal Human Ethics Committee and by the Regroupement Neuroimagerie/Québec (RNQ). This committee follows the guidelines of the Civil Code of Quebec, the Tri-Council Policy Statement of Canada, the Declaration of Helsinki, and the code of Nuremberg.

**Table 1:** Means (M) and standard deviations (SD) of the demographic and neuropsychological variables of all participants (*n* = 40).

	**Younger (*n* = 20)**	**Older (*n* = 20)**		
	**M (SD)**	**M (SD)**	***F*_(1.38)_**	***p*-values**
Age	24.85 (3.85)	69.45 (4.54)	1129.02	<0.001
Gender (F: M)	16:4	17:3	0.603	0.714
Education (years)	17.95 (2.52)	18.85 (2.88)	1.01	0.301
Edinburgh inventory	95%	96%	0.89	0.122
MoCA	28.6 (1.53)	28.7 (1.03)	0.058	0.81
Stroop C (seconds)	49.95 (6.88)	62.2 (9.12)	22.95	<0.001
Stroop W (seconds)	39.25 (4.02)	45.30 (5.82)	14.61	<0.001
Stroop C–W (seconds)	83.05 (13.42)	114.65 (22.29)	29.5	<0.001
TMT A (seconds)	17.40 (4.35)	27.60 (8.22)	23.98	<0.001
TMT B (seconds)	41.60 (11.77)	62.82 (16.29)	22.24	<0.001
TMT B-A (seconds)	24.20 (2.5)	34.90 (3.53)	6.08	<0.05
Digits forward	10.5 (1.67)	9.65 (1.75)	2.46	0.125
Digits backward	8.45 (2.03)	6.8 (1.73)	7.6	<0.01
Brixton (errors)	1.15 (1.03)	1.45 (1.05)	0.82	0.37
WCST (errors)	0.88 (1.19)	1.05 (1.27)	0.41	0.527
Semantic Fluency (2 min)	39.70 (8.27)	28.85 (8.1)	17.55	<0.001

*SD, Standard deviation*.

#### Stimuli selection

The experimental task required participants to sort words according to different levels of semantic relatedness. In order to construct the task, 169 stimulus words were chosen from the databases created by Dubois and Reshe-Rigon (1995) and Léger et al. (2008). The category “animals” was selected (subcategories: birds, insects, quadrupeds, and fish). Before the final stimuli were presented to participants, a pilot study was carried out in which 40 volunteers (20 younger, *M* = 26.92 years; SD = 6.02 and 20 older, *M* = 60 years, SD = 5.75) were asked to identify the items they had never heard before in a list of words. Based on this assessment, items that were identified as unknown by three or more participants were excluded.

**Figure 1:**
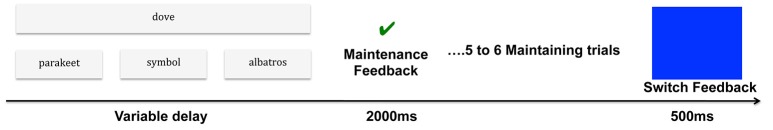
Experimental procedure of the word-matching task. In this example, each participant performed a task in which a target word presented at the top of the screen, dove (“*colombe*”) had to be paired with one of three reference words, presented at the bottom, according to three possible semantic relationships: (a) typically related word (co-hyponyms) parakeet (“*perruche*”); (b) atypically related word (co-hyponyms) albatross (“*albatross*”); and (c) functionally related words (F) symbol (“*symbole*”). The sorting period was followed by a maintenance feedback signal (green checkmark displayed for 2,000 ms) indicating that participants should repeat the application of the same semantic rule as in the previous trial. After 5 or 6 correct same rule application trials, the rule changed (blue screen displayed for 500 ms) and participants had to discover the new classification rule and maintain it.

Afterwards, two types of semantic relatedness were also measured: typicality relatedness and functional relatedness. Two groups of 20 participants each (20 younger and 20 older) were asked to estimate the extent to which these words represented a category on a 7-point scale (1 = least typical; 7 = most typical). First, the typical and atypical words were selected following the consensus obtained by the two groups (Typical: younger: mean = 5.67, SD = 0.09; older: mean = 5.58, SD = 0.10; Atypical: younger: mean = 3.77, SD = 0.1; older: mean = 3.75, SD = 0.99). Then, from this selection, the moderately and highly typical words were selected and divided into two lists. The highly typical words were chosen as target words and presented to the same sample of participants in order to establish a list of functionally related words. For the last step in stimulus selection of functionally related words, a similar pilot study assessed the extent to which five words (Figure 1 databases of Nelson et al., 2004; De Deyne and Storms, 2008) were strongly associated with each highly typical target word. All participants were instructed to read each highly typical word and then write down the first five associated words that immediately came to their minds. They were encouraged to respond as quickly as possible. However, no time limit was imposed to participants. The dominant response, i.e., the one produced by the largest number of participants, was chosen as the expected response for target words.

The final list contained 40 highly typical words (i.e., the target words), 40 moderately typical words, 40 atypical words and 40 functionally related words. None of the pilot participants were invited to participate in the experimental word-matching task. All stimuli were controlled for frequency, imageability and word length with the French lexical database Lexique 3 (New et al., 2005; www.lexique.org). In terms of lexical frequency a significant difference was found between functional and atypical (*M* = 5.15, SD = 1.3; *M* = 1.9; SD = 0.27; *p* = 0.016) and between moderately typical and atypical (*M* = 3.2, SD = 0.5; *M* = 1.9; SD = 0.27; *p* = 0.037). No significant difference was found between functional and moderately typical (*M* = 5.15, SD = 1.3; *M* = 5.42, SD = 1.18; *p* = 0.142). In terms of degree of imageability a significant difference was found between functional and atypical (*M* = 5.7, SD = 0.13; *M* = 4.9, SD = 0.17; *p* = 0.002) and between moderately typical and atypical (*M* = 5.42, SD = 0.18; *M* = 4.9, SD = 0.17; *p* = 0.081). No significant difference was found between functional and moderately typical (*M* = 5.7, SD = 0.13; *M* = 5.42, SD = 0.18; *p* = 0.384). In terms of word length, no significant difference was found between the three reference words [functional (*M* = 6.80, SD = 1.34), moderately typical (*M* = 6.60, SD = 1.49), atypical (*M* = 6.60, SD = 1.53), *p* = 0.78].

#### Task procedure

The word-matching task used in this study was based on the computerized WCST developed and adapted to fMRI by Monchi et al. (2001) and Simard et al. (2011). The word-matching task was administered using stimulus presentation software (Media Control Function; Digivox, Montréal, Canada). Throughout the task, three reference cards based on three semantic rules were presented in a row at the bottom of the screen, displaying moderately, atypical, and functionally related words (see Figure 1 for example). In each trial, a new target card was presented in the middle of the screen above the reference cards; it displayed a highly typical word. Participants must then match the target card with one of the reference cards based on moderately typical, atypical, or functional relatedness. Participants used a joystick to select among the three reference words, pressing left, right, or upward to select the reference word on the left, on the right, or in the middle, respectively.

The word-matching task trials contained two periods: matching and feedback.

- The matching period started with the presentation of a new target card (highly typical word). The participant then chose one of the three reference words by using one of the three joystick directions. The length of each matching period depended on the participant's response times, which varied between 1,470 and 4,690 milli-seconds (ms) for this task. The period ended when the participant provided a selection response.
- The feedback period was indicated by a blue screen, which lasted for 500 ms and started as soon as a first correct match was made. Feedback was conveyed through a specific cue lasting for 2,000 ms. An incorrect match was indicated by a red cross, whereas a correct match was indicated by a green check mark, which informed participants that the current matching rule was the correct one and that they should maintain the same rule as in the previous trial (see Figure 1 for experimental procedure).
- In addition, there were control trials during which the target card was represented by a series of letters (e.g., AAAA), which was identical with one of the three reference cards (e.g., aaaa, bbbb, cccc). These trials involved pairing a target with an identical reference card (alphabetic association: AAAA with aaaa). No rule changes occurred in the control condition and control feedback indicated a correct or incorrect match.

All participants had one fMRI session, which consisted of four runs. Blocks of each of the four trials (the three semantic rule trials and the control trial) were presented in pseudo-random order four times per run. The rules changed without warning and the new correct rule would be applied and maintained until the participant achieved five to six consecutive correct matching trials (maintaining a rule if shown a green check mark) or had to switch it (if presented with a blue screen as feedback). It is worth mentioning that no participant reported learning the sequence regularity or having deduced the frequency of the changing rule. The control block consisted of eight trials. For each participant, the total number of trials per run changed according to performance, which depended on the number of errors. The participants were fully trained on the word-matching task by performing a block of conditions outside the scanner. Each participant needed to reach a performance level of 90% correct matching trials and have <5% of set-loss and perseverative errors before moving on to the scanning session.

The stimuli were presented via an LCD projector onto a mirror placed in front of the participant in the MRI scanner. Stimuli were outlined in black against a white background to improve visual contrast (Figure 1). All words were displayed horizontally at the top of the screen and were centered on a computer screen placed 50 cm away from the participant. The target word was placed in a larger rectangle and subtended a visual angle of 26.6° horizontally and 13.8° vertically. All words were presented in 28-point Arial font, and reference words were placed in three small rectangles 1.3 cm apart from each other.

With regard to the study purpose, only the correct (5–6) consecutive matching trials after the maintenance feedback period (henceforward referred to as *rule repetition*, for each semantic condition), was taken into account for behavioral and imaging analysis. Furthermore, to ensure that the new semantic rule was successfully acquired after a rule-matching change (related to the search for a correct rule), the first correct matching trial after switch feedback was removed. Several contrasts were generated for analysis by subtracting the control matching condition from the rule repetition condition for each of the three semantic rules as well as by subtracting the repetition of one semantic relationship type from another one. These contrasts are (1) repetition of the functional rule minus control condition; (2) repetition of the typical rule minus control condition; (3) repetition of the atypical rule minus control condition; (4) repetition of the functional rule minus typical rule; (5) repetition of the typical rule minus functional rule (6) repetition of the atypical rule minus typical rule; (7) repetition of the typical rule minus atypical rule. It should be noted that analysis performed for the contrast 5 and 7 did not show significant activation difference and for this reason are not reported in the present manuscript. For ease of description, and the pivotal interest of neuroimaging findings in healthy aging, only direct contrast comparison between older compared to the younger are reported in this manuscript. Nevertheless, the reverse contrast (younger compared to older) was also performed but did not elicit any significant difference in brain activation.

## fMRI scanning

### Image acquisition

Participants were scanned at the Unité de Neuroimagerie Fonctionnelle of the Institut de Gériatrie de Montréal using a 3T Siemens Trio Magnetom MRI scanner (Siemens AG, Erlangen, Germany). The structural scan was a high-resolution T1-weighted 3D-MPRAGE, sagittal plane acquisition, field of view (FOV) = 256 mm, and matrix size = 256 × 256. In addition, we acquired functional images (T2^*^ weighted, TR = 2,500 ms, TE = 30 ms, 36 slices parallel to the anterior and posterior commissure (AC-PC) line, slice thickness = 3.5 mm with 3.5 mm^3^ isotropic voxels, distance factor 0% (gap = 0 mm), Flip-angle = 90°, matrix = 64 × 64). Each 252-volume functional run lasted 10.5 min; four such runs were acquired for each participant. The stimulus presentation and the scanning were synchronized at the beginning of each run. To minimize head movement during scanning, cushions were placed between the subject's head and the coil.

### Data analysis

FEAT (FMRI Expert Analysis Tool) Version 5.98, part of the FSL analysis package (FMRIB's Software Library, Version 4.1.4^1^), was used to conduct image pre-processing procedures. We corrected for head motion using MCFLIRT (FMRIB's motion correction linear image registration tool; Jenkinson and Smith, 2001), and also used the fsl_motion_outliers script to detect and remove any volumes with excessive head motion. Non-brain tissue was removed using Brain Extraction Tool (BET; Smith, 2002). Grand-mean intensity normalization was applied to the 4D dataset from each run based on multiplicative scaling factor. We applied a Gaussian kernel of 6 mm FWHM for spatial smoothing, and for temporal filtering, a high-pass filter was applied to remove low-frequency noise using Gaussian-weighted least-squares straight-line fitting (1/60 Hz). Temporal auto-correlation was corrected by using pre-whitening as implemented by FILM (FMRIB's improved linear model). Functional images of each participant were co-registered to structural images in native space, and structural images were normalized to Montreal Neurological Institute (MNI) standard space using FSL's MNI Avg 152 T1 2 × 2 × 2 mm. The same transformation matrices used for structural-to-standard transformations were then used for functional-to-standard space transformations of co-registered functional images.

The FEAT module in FSL was used for first level analysis. An event-related design was used to model the fMRI data, allowing for inference based on contrast. We included four different event types in the design matrix: functional, typical, and atypical rules; and control trials. The rule repetition period was defined on the basis of the time period, for which each length varied between trials depending on the participant's response time. This period started with the presentation of a new trial and ended only when participant provided a selection response. The rule repetition period was convolved with a double-gamma hemodynamic response function (HRF). The aim was to explore all consecutive correct repetition rule periods for each semantic relationship (functional, typical, and atypical).

A first-level GLM analysis was carried out separately for each run, including extended motion regressors generated from MCFLIRT estimates as confound variables. A between-subject GLM analysis was performed on first-level betas across the four runs to test for main-effect and age group brain activation differences during each semantic rule period (Functional, Typical and Atypical). Non-parametric statistical inference was applied with FSL Randomize (Nichols and Holmes, 2002) to correct for voxel-wise multiple comparisons and cluster-size, using threshold-free cluster enhancement (TFCE, Smith and Nichols, 2009). The latter process consists of fitting the between-subject GLM model performed with *n* = 10,000 sign flipping, and group permutation for main and group effects respectively, in order to generate a null distribution of both voxel-wise statistics and cluster-size. The resulting TFCE maps were at a threshold of *p* < 0.05 for display and extraction of clusters.

Behavioral data (response times and correct responses) were averaged for each group and for each of the three type of semantic rule (Functional vs. Typical vs. Atypical). A 3 × 2 ANOVA (semantic rules x age group) was performed using SPSS 15.0 for Mac. A comparison between the two groups for each semantic rule and between semantic rules for each group was done by ANOVAs.

## Results

### Behavioral performance

#### Response times

We conducted a 3 × 2 analysis of variance with the level of semantic control demands (Functional vs. Typical vs. Atypical) and the age group (Younger vs. Older) on the response times for repeating the application of semantic rule in word matching task (Table 2). There was a main effect of age group [*F*_(1.38)_ = 8.397, *p* < 0.01] with response times being significantly longer in the older group than in the younger group. There was also significant main effect of semantic rule [*F*_(2.38)_ = 122.28, *p* < 0.001], reflecting an increase in response times with a level of semantic control demand (Functional < Typical < Atypical). A planned comparison of level of semantic control demand shows significant difference between younger and older adults [Functional: M_older_ = 2,234 ms, SD = 467; M_younger_ = 1,669 ms, SD = 390; *F*_(1.38)_ = 17.182, *p* < 0.001; Typical: M_older_ = 2888 ms, SD = 490; M_younger_ = 2,366 ms, SD = 755; *F*_(1.38)_ = 6.704, *p* < 0.05; Atypical: M_older_ = 3,103 ms, SD = 549; M_younger_ = 2,559 ms, SD = 949; *F*_(1.38)_ = 4.907, *p* < 0.05]. Furthermore, a comparison between different semantic rules showed that younger adults as well as older adults tend to be faster during repeating the application of functional rule compared both to typical and to atypical rule [Younger: *F*_(2.57)_ = 8.098, *p* < 0.001; Functional vs. Typical, *p* < 0.05; Functional vs. Atypical, *p* < 0.001; Older: *F*_(2.57)_ = 16.156, *p* < 0.001; Functional vs. Typical, *p* < 0.001; Functional vs. Atypical, *p* < 0.001].

**Table 2:** Behavioral performance (response times and correct responses) during rule repetition in word-matching task.

	**Younger (*n* = 20)**	**Older (*n* = 20)**		
	**Mean (SD)**	**Mean (SD)**	***F*_(1.38)_**	***p*-values**
**RESPONSE TIMES (IN ms)**				
Functional	1,669 (390)	2234 (467)	17.18	<0.001
Typical	2,366 (755)	2888 (490)	6.70	<0.05
Atypical	2,559 (949)	3103 (549)	4.90	<0.05
**CORRECT RESPONSES (IN %)**				
Functional	97.25 (2.60)	94.40 (3.75)	3.26	0.08
Typical	96.09 (2.20)	93.30 (4.25)	3.71	0.061
Atypical	95.32 (3.51)	91.48 (4.30)	15.18	<0.001

*SD, Standard deviation*.

#### Correct responses

The mean percentage of correct responses was considered as all correct consecutive repeating applications of the same semantic rule. A main effect of age group was found [*F*_(1.38)_ = 10.89, *p* < 0.01] with percentage of correct response being significantly lower in the older adults than in the younger adults. There was also a significant main effect of semantic rule [*F*_(2.38)_ = 3.505, *p* < 0.05], suggesting a decrease in percentage of correct responses with the level of semantic control demand. A planned comparison of level of semantic control demand shows significant difference of correct responses between younger and older adults only for rule repetition according to atypical condition (*p* < 0.001; Table 2). A comparison between semantic rules showed that the younger adults as well as the older adults tend to perform accurately during repeating the application of semantic rules [Younger: *F*_(2.57)_ = 1.191, *p* = 0.311; Older: *F*_(2.57)_ = 2.531, *p* = 0.42].

### Imaging results

#### Level of semantic control demands

**Figure 2:**
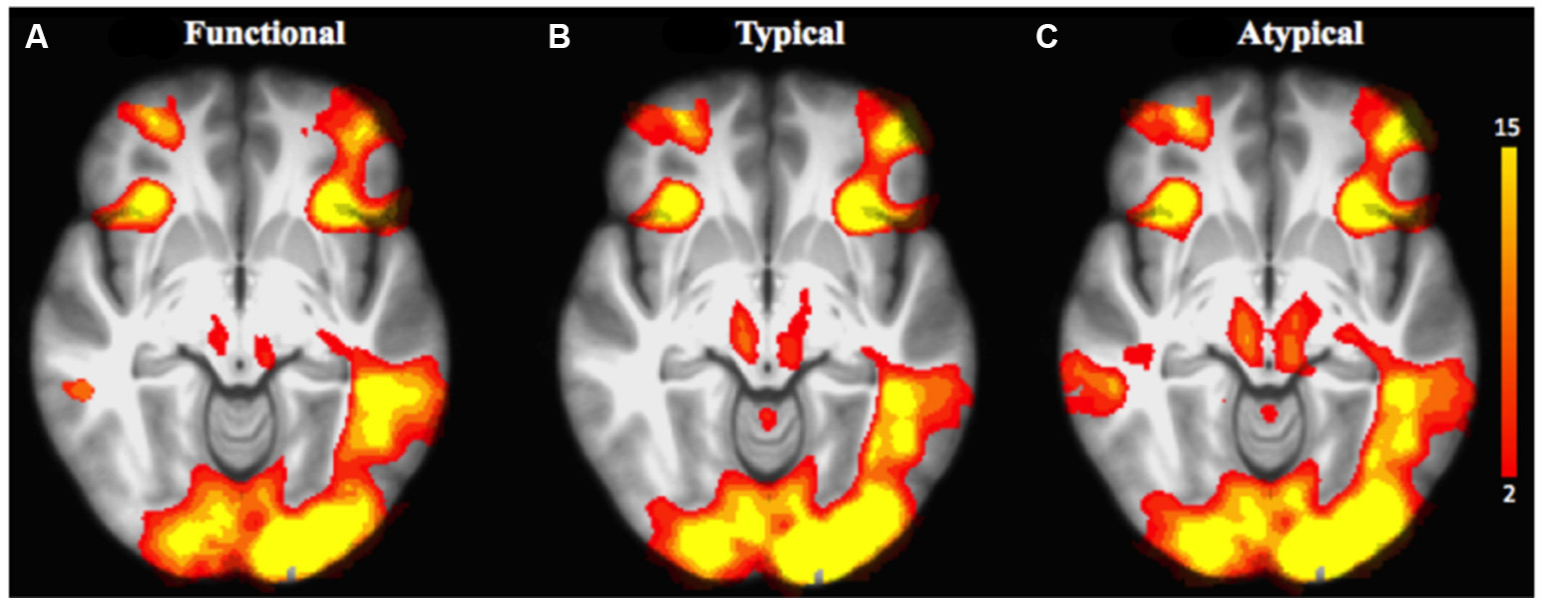
Brain activation related to the repetition of the application of the functional rule (cf. **A**), typical rule (cf. **B**), and atypical rule (cf. **C**) relative to control condition for all age groups. The scale illustrated the value of the t-maps. Threshold-free Cluster Enhancement (TFCE) at *p* < 0.05.

**Figure 3:**
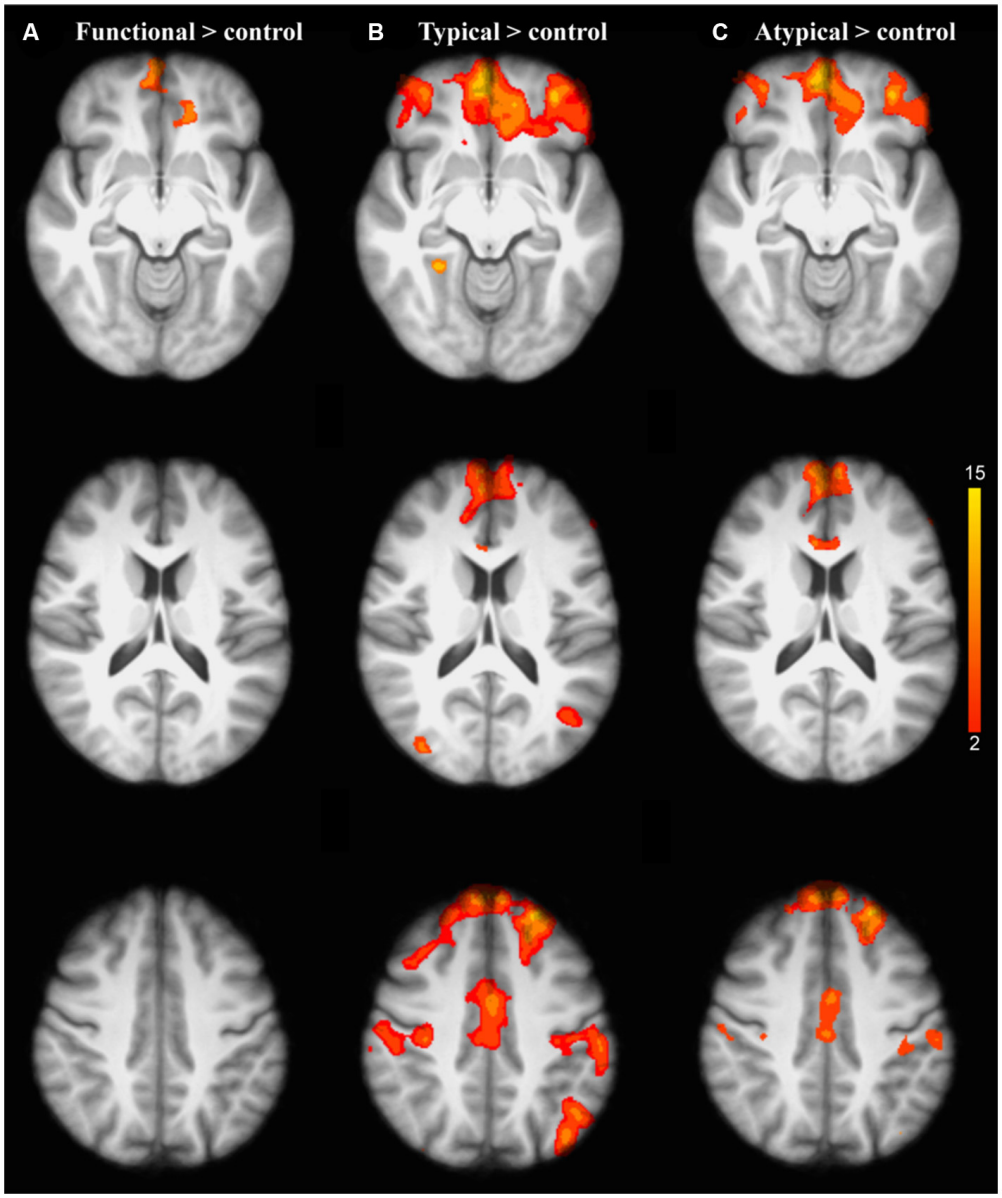
Brain activation related to the repetition of each semantic rule relative to control condition for older adults compared to the younger adults: **(A)** Functional; **(B)** Typical; **(C)** Atypical. The scale illustrated the value of the t-maps. Threshold-free Cluster Enhancement (TFCE) at *p* < 0.05.

We first explored task-related activation changes (Table 3, Figures 2A–C; two age groups together. We also investigated direct contrasts of brain activation between the two age groups (older compared to younger) associated to semantic control demands (Functional vs. Typical vs. Atypical) relative to control condition (Tables 4–**6**; Figures 3A–C). As predicted, the neuroimaging analysis revealed age- and task-related activation changes associated with the repetition of semantic rule (Functional vs. Typical vs. Atypical) confirming the semantic processing demand manipulation was related to neuro-functional change when faced with a challenge insofar as it reflects the recruitment of additional neural resources. Indeed, age and task-related significant activation differences associated to semantic control demands were found in several parts of semantic control networks.

**Table 3:** Significant activation clusters associated with the repetition of semantic rules (functional, typical, atypical) for all age groups.

**Cluster region**	**Cluster size**	***t*-value**	**MNI coordinates (x, y, z)**		
**FUNCTIONAL** > **CONTROL**					
Right cerebellum	594,706	12.3	9	−75	−28
Left insula cortex (area 13)	–	12.1	−31	21	−1
Left inferior parietal cortex (area 39, AG)	–	12.1	−31	21	−1
Right insula cortex (area 13)	–	11.9	32	25	1
Right occipital cortex (area 17)	–	11.1	15	−91	1
Left occipital cortex (area 17)	–	11.1	−13	−91	−4
Right inferior parietal cortex (area 39, AG)	–	9.51	35	−62	42
Right dorsal medial PFC (area 8)	–	9.48	35	−62	42
Left posterior PFC (area 44)	–	9.36	−44	17	25
Right mid-dorsolateral PFC (area 9)	–	9.02	49	35	25
Left inferior temporal cortex (area 37 FG)	–	8.48	−48	−56	−14
Left cerebellum	–	7.25	−25	−62	−31
Left dorsolateral PFC (area 46)	–	6.77	−50	44	12
Left posterior middle temporal cortex (area 21)	–	6.53	−52	−35	2
Right superior parietal cortex (area 7)	–	6.49	11	−74	57
Right thalamus	–	6.3	9	−3	1
Left orbitofrontal cortex (area 11)	–	5.64	−21	39	−22
Right lateral premotor cortex (area 6)	–	5.11	36	−2	39
Right frontopolar cortex (area 10)	–	4.99	37	53	24
Right caudate nucleus	–	4.24	19	−14	22
Left ventrolateral PFC (area 47)	–	4.18	−54	26	−5
Left putamen	–	3.54	−17	−2	−1
Left frontopolar (area 10)	–	2.94	−35	62	−9
Right posterior middle temporal cortex (area 21)	551	4.33	66	−31	−12
**TYPICAL** > **CONTROL**					
Left insula cortex (area 13)	640,006	14.8	−31	21	−1
Left occipital cortex (area 18)	–	14.6	−13	−94	−4
Right cerebellum	–	13.9	8	−76	−27
Left inferior parietal cortex (area 39, AG)	–	13.1	−28	−66	41
Right insula cortex (area 13)	–	13.1	32	24	1
Left posterior PFC (area 44)	–	12.5	−39	23	23
Right occipital cortex (area 18)	–	11.4	20	−93	−1
Right inferior parietal cortex (area 39, AG)	–	10.9	34	−62	42
Left cerebellum	–	10.5	−39	−67	−29
Right dorsolateral PFC (area 9/46)	–	9.96	51	31	28
Left lateral premotor cortex (area 6)	–	9.86	−36	1	34
Left inferior temporal cortex (area 37 FG)	–	9.48	−48	−49	−14
Left dorsolateral PFC (area 46)	–	7.99	−48	47	1
Right superior parietal cortex (area 7)	–	7.4	12	−75	57
Left orbitofrontal cortex (area 11)	–	6.5	−21	−77	14
Right lateral premotor cortex (area 6)	–	6.16	35	4	58
Right frontopolar cortex (area 10)	–	5.62	24	48	−15
Left globus pallidus	–	5.06	−17	−3	−3
Left superior temporal cortex (area 22)	–	4.93	−57	−37	6
Left frontopolar cortex (area 10)	–	4.93	−37	54	24
Left dorsal medial PFC (area 8)	–	4.28	31	13	34
Left ventrolateral PFC (area 47)	–	4.08	−25	22	−26
Right dorsal medial PFC (area 8)	–	3.04	−34	33	48
**ATYPICAL** > **CONTROL**					
Left insula cortex (area 13)	728,734	15.5	−29	22	0
Right insula cortex (area 13)	–	15.1	32	25	1
Right dorsal medial PFC (area 8)	–	14.1	5	26	44
Right cerebellum	–	13.6	9	−77	−27
Left inferior parietal cortex (area 39, AG)	–	13.5	−28	−66	42
Left occipital cortex (area 18)	–	13.4	−13	−94	−3
Left posterior PFC (area 44)	–	12.9	−39	23	23
Right inferior parietal cortex (area 39, AG)	–	12.1	33	−61	45
Right occipital cortex (area 18)	–	11.9	20	−94	−2
Left cerebellum	–	11	37	−66	−28
Right mid-dorsolateral PFC (area 9)	–	10.8	53	36	24
Left lateral premotor cortex (area 6)	–	9.94	−35	0	34
Right posterior cingulate cortex (area 23)	–	9	29	−63	10
Left frontopolar cortex (area 10)	–	8.39	−45	54	1
Right superior parietal cortex (area 7)	–	8.27	12	−75	58
Left inferior temporal cortex (area 37, FG)	–	8.17	−48	−49	−13
Right globus pallidus	–	7.66	15	0	−2
Right lateral premotor cortex (area 6)	–	6.88	33	4	54
Right posterior PFC (area 44)	–	6.76	41	13	26
Left orbitofrontal cortex (area 11)	–	6.05	−21	49	−16
Right inferior parietal cortex (area 40, SM)	–	5.83	48	−41	46
Right frontopolar cortex (area 10)	–	5.72	24	47	−16
Left thalamus	–	5.55	−20	−33	4
Right caudate nucleus	–	5.45	19	−12	22
Right posterior middle temporal cortex (area 21)	–	4.75	54	−40	−8
Left globus pallidus	–	4.51	−17	−4	−3
Left superior parietal cortex (area 7)	–	4.18	−12	−72	62
Left ventrolateral PFC (area 45)	–	3.42	−53	20	−2

*AG, Angular gyrus; PFC, prefrontal cortex; FG, fusiform gyrus; SM, supramarginal gyrus*.

**Table 4:** Significant activation clusters associated to repetition of functional rule relative to control condition for older minus younger adults.

**Cluster region**	**Cluster size**	***t*-value**	**MNI coordinates (x, y, z)**		
Right frontopolar cortex (area 10)	4,000	4.49	2	62	−11
Left orbitofrontal cortex (area 11)	–	4.3	−16	37	−10
Right ventrolateral PFC	349	5.31	25	19	−22
Left frontopolar cortex (area 10)	145	3.85	−3	66	6

*PFC, Prefrontal cortex*.

##### Task-related neuro-functional changes (all age groups together)

For the comparison of each experimental condition (functional, typical, and atypical) with control condition (main task-effect), task-related activation changes were found, owing to semantic control demands (Table 3, Figures 2A–C). As predicted, significantly larger activation clusters were found in the higher level of semantic control demand represented by the atypical rule compared to typical and functional rules. This finding revealed that the semantic control network was differentially engaged depending on task demand as well as by unique functional contributions by specific brain regions within this network.

Indeed, significant activation clusters were consistently found by all experimental conditions regardless of whether the type of semantic rule is highly functional or more categorical (typical vs. atypical), when control condition was subtracted from experimental condition. This task-related activation (all age groups together) was found at the cerebellum bilaterally, the insula cortex (area 13), the inferior parietal cortex (area 39, angular gyrus), the occipital cortex (area 17) and the frontopolar cortex (area 10). However, patterns of activation associated to atypical rule were found to be more widespread than those for typical and functional.

Functional-related activation difference was found in the right dorsal medial PFC (area 8), the left posterior PFC (area 44), the right mid-dorsolateral PFC (area 9), the left inferior temporal cortex (area 37), the left dorsolateral PFC (area 46), the posterior middle temporal cortex bilaterally (area 21), the right superior parietal cortex (area 7), the right orbitofrontal cortex (area 11), the right lateral premotor cortex (area 6), and the left ventrolateral PFC (area 47). Subcortically, significantly greater activation was found in the right thalamus, caudate nucleus and in the left putamen.

Typical rule-related activation difference was found bilaterally in the dorsolateral PFC (area 9/46, 46), the left posterior PFC (area 44), the left lateral PFC (area 6), the left inferior temporal (area 37), the right superior parietal cortex (area 7), the left orbitofrontal (area 11), the left superior temporal cortex (area 22), the dorsal medial PFC bilaterally (area 8), the left ventrolateral PFC (area 47). Subcortically, significantly greater activation was found in the left globus pallidus.

In addition, differences in activation were also found for atypical rule repetition in additional brain regions including the right dorsal medial PFC (area 8), the posterior PFC (area 44) bilaterally, the right mid-dorsolateral PFC (area 9), the lateral premotor cortex (area 6) bilaterally, the right posterior cingulate cortex (area 23), the superior parietal cortex bilaterally (area 7), the left inferior temporal cortex (area 37), the left orbitofrontal cortex (area 11), the right inferior parietal cortex (area 40, supramarginal gyrus), the right posterior middle temporal cortex (area 21), and the left ventrolateral PFC (area 45). Subcortically, significant activation was observed bilaterally in the globus pallidus, the left thalamus, and in the right caudate nucleus.

##### Age-related neuro-functional changes

As predicted, the analysis of the interaction between task and age group revealed that the repetition of the application of a given semantic rule was driven by significant brain activation changes in healthy aging. Indeed, the older adults showed significant activation changes compared to the younger ones in the inferior prefrontal regions (frontopolar, orbitofrontal, and ventrolateral PFC) revealing graded differences in the repetition of the application of a given semantic rule (Figures 3A–C).

For the inter-group comparison (older minus younger), the older adults showed significant activation changes compared to the younger adults for each semantic condition:

*When functional was compared to the control condition* (Table 4), significant activation of clusters was found in the frontopolar bilaterally (area 10), the left orbitofrontal cortex (area 11) and in the right ventrolateral PFC (area 47).

*When typical was compared to control condition* (Table 5), larger significant activation clusters were found bilaterally in the frontopolar cortex (area 10) as well as in the orbitofrontal cortex (area 11) and the ventrolateral PFC (area 47). Additionally, older adults (compared to the younger) further recruit additional brain regions including, the left dorsolateral PFC (area 46), the right dorsal medial PFC (area 8), the left lateral premotor cortex (area 6), the left superior parietal cortex (area 7), the left inferior parietal cortex (area 39 and 40, angular and supramarginal gyri, respectively), the left anterior cingulate cortex (area 24), the left posterior cingulate and the right occipital cortex (area 18).

**Table 5:** Significant activation clusters associated to repetition of typical rule relative to control condition for older minus younger adults.

**Cluster region**	**Cluster size**	***t*-value**	**MNI coordinates (x, y, z)**		
Right frontopolar cortex (area 10)	96,794	5.42	5	53	−8
Left frontopolar cortex (area 10)	–	5.33	−10	62	23
Left orbitofrontal cortex (area 11)	–	4.98	−7	32	−16
Right ventrolateral PFC (area 47)	–	4.44	39	31	−20
Left dorsolateral PFC (area 46)	–	4.25	−55	38	6
Right orbitofrontal cortex (area 11)	–	3.9	16	20	−19
Left ventrolateral PFC (area 47)	–	3.8	−33	20	−20
Right dorsal medial PFC (area 8)	–	3.12	41	19	41
Right lateral premotor cortex (area 6)	93,147	5.29	21	−8	57
Left superior parietal cortex (area 7)	–	5.17	−25	−64	69
Left precentral (area 4)	–	5.07	−21	−16	63
Left inferior parietal cortex (area 39, AG)	–	4.86	−40	−71	38
Left anterior cingulate cortex (area 24)	–	4.62	−1	−8	45
Left inferior parietal cortex (area 40, SM)	–	4.17	−60	−36	40
Left posterior cingulate cortex (area 23)	–	2.44	−12	−29	36
Right occipital cortex (area 18)	1,582	4.14	37	−82	20

*AG, Angular gyrus; PFC, prefrontal cortex; SM, supramarginal gyrus*.

*When atypical was compared to control condition* (Table 6), significant activation difference was observed bilaterally in the frontopolar (area 10), the orbitofrontal (area 11), the ventrolateral PFC (area 45 and 47), the left superior parietal cortex (area 70, the left inferior parietal cortex (area 39). In prefrontal cortex, bilaterally significant clusters in the dorsal medial PFC (area 8) and the lateral premotor cortex (area 6) were more recruited by older adults.

**Table 6:** Significant activation clusters associated to repetition of atypical rule relative to control condition for older minus younger adults.

**Cluster region**	**Cluster size**	***t*-value**	**MNI coordinates (x, y, z)**		
Right frontopolar cortex (area 10)	49,815	5.39	0	57	−2
Left frontopolar cortex (area 10)	–	5.32	−10	62	23
Left dorsal medial PFC (area 8)	–	5.28	−26	38	48
Right dorsal medial PFC (area 8)	–	4.97	5	51	45
Right ventrolateral PFC (area 47)	–	4.87	40	32	−21
Left orbitofrontal cortex (area 11)	–	4.56	−7	32	−15
Right orbitofrontal cortex (area 11)	–	4.1	17	20	−20
Left lateral premotor cortex (area 6)	16,275	4.9	−19	−20	63
Left ventrolateral PFC (area 45)	8,908	5.1	−55	39	−5
Right posterior cingulate cortex (area 31)	3,825	4.54	1	−26	47
Right lateral premotor cortex (area 6)	1,747	5.7	21	−8	58
Left superior parietal cortex (area 7)	346	4.11	−22	−61	69
Left inferior parietal cortex (area 39, AG)	327	4.49	−40	−71	38

*AG, Angular gyrus; PFC, prefrontal cortex*.

#### Inter-semantic rules comparisons

**Figure 4:**
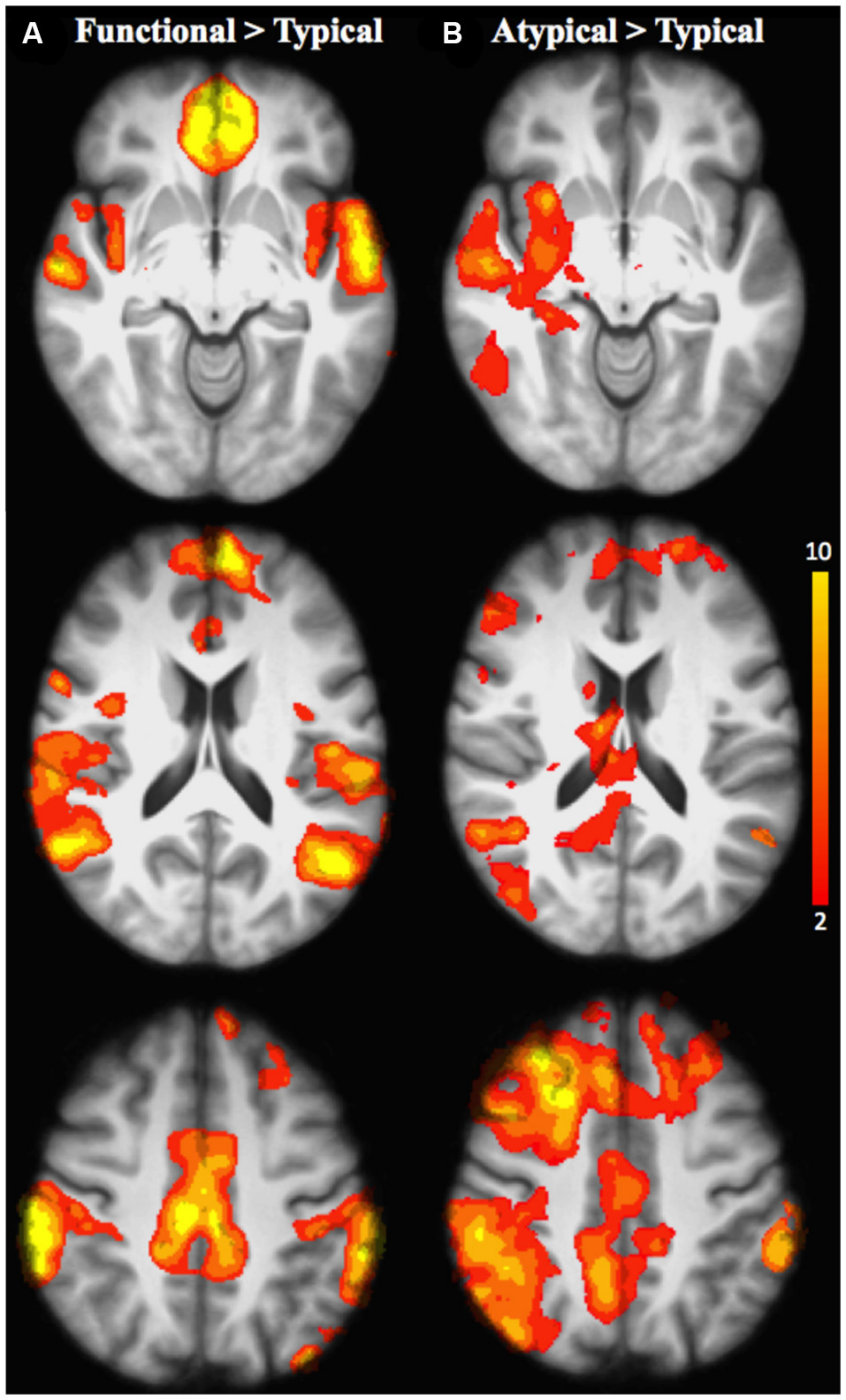
Brain activation for the inter-semantic rules comparison in all age groups: **(A)** Functional relative to Typical rule; **(B)** Atypical relative to Typical rule. The scale illustrated the value of the t-maps. Threshold-free Cluster Enhancement (TFCE) at *p* < 0.05.

Differences in terms of brain activation changes for inter-semantic rules comparison based on the functional and categorical relationship (Functional vs. Typical and Atypical vs. Typical) are shown in Tables 7, 8, Figures 4A,B.

**Table 7:** Significant activation clusters associated to repetition of functional rule relative to typical rule.

**Cluster region**	**Cluster size**	***t*-value**	**MNI coordinates (x, y, z)**		
Left frontopolar cortex (area 10)	264,446	10.3	−4	59	−1
Left inferior parietal cortex (area 40, SM)	–	8.04	−59	−38	38
Left superior temporal cortex (area 22)	–	7.57	−59	−4	−9
Left inferior parietal cortex (area 39, AG)	–	6.76	−49	−63	23
Right posterior cingulate (area 31)	–	6.66	8	−24	41
Left temporopolar cortex (area 38)	–	6.43	−45	18	−31
Right orbitofrontal cortex (area 11)	–	6.43	9	35	−7
Left insula cortex (area 13)	–	6.22	−34	5	8
Left anterior cingulate cortex (area 32)	–	5.86	−2	7	37
Left superior parietal cortex (area 7)	–	5.05	−24	−44	75
Left mid-dorsolateral PFC (area 9)	–	4.38	−23	28	33
Right mid-dorsolateral PFC (area 9)	–	4.08	17	52	28
Left lateral premotor cortex (area 6)	–	3.88	−8	−10	60
Left dorsal medial PFC (area 8)	–	3.73	−12	48	48
Left superior temporal cortex (area 22)	–	3.23	−67	−38	14
Left posterior middle temporal cortex (area 21)	–	2.65	−49	−37	−2
Right inferior parietal cortex (area 40, SM)	137,227	7.8	60	−32	31
Right temporopolar cortex (area 38)	–	6.85	51	20	−26
Right inferior parietal cortex (area 39, dorsal AG)	–	6.5	57	−55	12

*AG, Angular gyrus; PFC, prefrontal cortex; SM, supramarginal gyrus*.

**Table 8:** Significant activation clusters associated to repetition of atypical rule relative to typical.

**Cluster region**	**Cluster size**	***t*-value**	**MNI coordinates (x, y, z)**		
Right dorsal medial PFC (area 8)	525,803	8.08	29	25	37
Right inferior parietal cortex (area 39, AG)	–	5.08	54	−60	32
Right inferior parietal cortex (area 40, SM)	–	5.61	61	−34	51
Right posterior cingulate (area 31)	–	5.47	11	−50	42
Right mid-dorsolateral PFC (area 9)	–	5.43	44	41	29
Right superior parietal cortex (area 7)	–	5.34	2	−71	63
Left inferior parietal cortex (area 40, SM)	–	5.12	−58	−43	43
Left anterior cingulate (area 23)	–	4.92	5	−7	41
Right putamen	–	4.9	28	11	−8
Left mid-dorsolateral cortex (area 9)	–	4.84	−32	36	35
Right superior temporal cortex (area 22)	–	4.84	55	−13	−9
Left inferior parietal cortex (area 39, AG)	–	4.8	−57	−56	21
Left frontopolar cortex (area 10)	–	4.79	−20	59	21
Right cerebellum	–	4.75	32	−79	−29
Left lateral premotor cortex (area 6)	–	4.58	−16	11	59
Right thalamus	–	4.43	9	−3	3
Right superior frontal cortex (area 6/8)	–	4.36	9	19	53
Right inferior temporal (area 37)	–	4.36	48	−57	−20
Right lateral premotor cortex (area 6)	–	4.27	31	9	65
Right postcentral (area 4)	–	4.18	25	−25	75
Right inferior temporal cortex (area 20)	–	3.94	42	−1	−39
Right frontopolar (area 10)	–	3.77	6	54	16
Right occipital cortex (area 19)	–	3.75	38	−86	15
Left cerebellum	–	3.61	−29	−60	−35
Right posterior PFC (area 44)	–	3.41	56	19	10

*AG, Angular gyrus; PFC, prefrontal cortex; SM, supramarginal gyrus*.

##### Task-related neuro-functional changes

When functional was compared with typical rule repetition regardless of age groups (Table 7, Figure 4A), significantly stronger activation were found in the left frontopolar cortex (area 10), the inferior parietal cortex bilaterally (area 39 and 40, angular and supramarginal gyri, respectively), the superior temporal cortex bilaterally (area 22), the right posterior cingulate (area 31), the temporopolar bilaterally (area 38), the right orbitofrontal cortex (area 11), the left insula (area 13), the left anterior cingulate (area 32), the left superior parietal cortex (area 7), the mid-dorsolateral PFC (area 9), the left lateral premotor cortex (area 6), the dorsal medial PFC (area 8) and the left posterior middle temporal cortex (area 21). The reverse inter-rules comparison, i.e., typical > functional showed no significant activation difference.

*When the atypical was compared to the typical rule repetition* (Table 8, Figure 4B), significantly stronger activations were observed in the right dorsal-medial PFC (area 8), the inferior parietal cortex bilaterally (area 39 and 40, angular and supramarginal gyri, respectively), the right posterior cingulate cortex (area 31), the mid-dorsolateral PFC bilaterally (area 9), the right superior parietal cortex (area 7), the left anterior cingulate cortex (area 23), the right putamen, the right superior temporal cortex (area 22), the frontopolar cortex bilaterally (area 10), the right thalamus, the cerebellum bilaterally, the lateral premotor cortex (area 6), the right superior frontal cortex (area 6/8) the right inferior temporal cortex (area 37 and 20), the right occipital cortex (area 19) and the right posterior PFC (area 44). The reverse contrast i.e., typical > atypical showed no significant activation difference.

##### Age-related neuro-functional changes

No significant difference in activation was observed for the interaction between task-and age group (older minus younger or younger minus older).

## Discussion

The aim of this study was to explore the age-related changes in patterns of brain activation underlying repetition of the application of a given semantic rule, using a word-matching task requiring different levels of semantic control demands (functional vs. typical vs. atypical). The main results point to differences in both behaviorally and neurofunctionally for the most semantic-control-demanding condition (atypical) for both younger and older participants (Table 2). However, even if performance was similar between younger and older participants, the latter group showed distinctive activation patterns. There was indeed a greater involvement of frontal regions for older adults in response to the increased demands, but this tended to decrease after having reached a certain level of high demand.

Regarding the first main result, both response times and the number of correct responses suggest that the implementation of an atypical rule of word-matching was a more difficult condition than typical and functional rule conditions for older and younger adults. This first result confirms that the different levels of semantic control demand we thought would be required for the application of the different semantic rules were indeed associated with distinct levels of cognitive difficulty. Consequently, this first result confirmed that the atypical rule does represent the most complex of the three rules used in our protocol. Of interest is how older adults responded more slowly and less accurately than younger adults for the repetition of the application of rules based on atypical relationship, and this despite also maintaining a relatively high level of correct responses (>90%), comparable to that of their younger counterparts. This finding is in line with previous behavioral studies of semantic categorization (Roskies et al., 2001; Khateb et al., 2003; Lei et al., 2010; Wang et al., 2016) showing that word matching is executed faster when participants are asked to identify a close semantic relationship between two words than when this relationship is more distant. Logically, typical words within a category share more semantic features than atypical ones. The latter condition can then be considered more “complex,” the matching execution relying on more variable features, and would require higher semantic control. Automatic spreading of activation in semantic memory would facilitate the retrieval of features, as well as the rapid and less effortful identification of pairs of more prototypical concepts in a given category; meanwhile, additional control resources will be required to match pairs of words linked by a smaller number of shared semantic features, thus resulting in a longer time required to match the two less prototypical words of a given category (Jackson et al., 2015). In this respect, Lambon Ralph et al. (2016) suggest that semantic cognition is underpinned by the interaction of two components: semantic representations and executive-control process. The latter process plays a pivotal role in controlled retrieval of semantic information, such that relevant aspects of meaning are brought to the foreground. Converging neuroimaging findings provide evidence of a widely distributed neural network supporting semantic representation and executive-control processes (Whitney et al., 2011; Noonan et al., 2013). These two processes seem to contribute conjointly in executive-demanding semantic tasks (Whitney et al., 2011; Fedorenko et al., 2013; Davey et al., 2016). Consequently, and consistent with our first prediction, *the first main finding* reported here shows differences in the extent of activations in response to increased task demands within the semantic control network (Table 3). The repetition of the application of an atypical rule is associated with large activations in areas that have been shown consistently to be involved in high semantic control demands condition (Figure 2). Executive control over semantic processing thus appears to be supported by a common and distributed neural network including bilateral prefrontal cortex PFC (frontopolar, orbitofrontal, ventro-lateral PFC), inferior parietal cortex, insula, and extending posteriorly in the left superior temporal cortex as well as bilaterally in the occipital and cerebellum. Although these task-related activation clusters are found to be larger during the repetition of the application of atypical rule than for typical and functional rules, additional right lateralized activation is also observed in other parts of network including posterior prefrontal regions and subcortical regions (i.e., caudate nucleus). The activation of the prefrontal, parietal and posterior temporal regions associated with semantic control across different semantic rules in the present study is indeed consistent with the view that these regions are of fundamental relevance in executive control across a wide range of cognitive domains (Noppeney et al., 2004; Whitney et al., 2009). Moreover, in the context of rule repetition as used in the present study, the PFC accounts largely for organizing goal-directed behavior, maintaining a previous task for subsequent retrieval and execution (frontopolar cortex; Hyafil and Koechlin, 2016), in reward-associated pair learning (orbitofrontal cortex, Robbins and Roberts, 2007), as well as for verbal rule acquisition and active retrieval from memory according to a rule (ventrolateral prefrontal cortex, Petrides and Pandya, 2002; Simard et al., 2011).

The activations reported herein for the inferior frontal area are therefore consistent with neurofunctional organization of the semantic/executive system, providing evidence that ventral aspects (i.e., ventrolateral, BA 47; frontopolar BA 10; and orbitofrontal, BA11) contribute to controlled semantic retrieval, while selection is more sustained by the dorsal inferior frontal cortex (dorsolateral prefrontal, BA 46).

In line with our results, and as proposed by Davey et al. (2016), semantic control appears to be supported by at least two processes: (a) First, a domain-general executive control sustained by a multiple domain network that allows for the goal-driven dimensions (application and maintenance) of the task (e.g., feature-matching task; Duncan, 2010); (b) Second, and automatic, an activation between strongly associated concepts within semantic system, independently from the executive control. Thus, the brain areas involved in semantic control as well as in the multiple-domain network would include dorsolateral prefrontal cortex, dorsal inferior frontal cortex, premotor cortex, parietal cortex and the posterior middle temporal cortex, and also the lateral occipital cortex. Given what is known about these areas, it is suggested that they are associated with the top-down allocation effort applied for task-demands.

A recent neuroimaging meta-analysis (Noonan et al., 2013) summarizes the brain regions within a semantic control network across many tasks requiring executive-semantic processing. The meta-analysis points to bilateral activations in the ventral and dorsal PFC, inferior parietal cortex, posterior middle temporal cortex, and anterior cingulate cortex. For instance, when high-control semantic processing is required to resolve the ambiguity of accessing less frequent meanings of words, functional coupling has been observed in the PFC and the posterior middle temporal cortex. Moreover, these two brain regions also appear to be consistently involved in executively demanding goal-oriented tasks across cognitive domains, suggesting that they represent a non-specific “multiple-demand network” (Davey et al., 2016). This suggests that the neural network supporting executive processing in the semantic domain overlaps with domain-general executive control. Beyond the contribution of the inferior prefrontal cortex in semantic control demand, there is also evidence for the additional contribution of inferior parietal cortex (angular gyrus) and posterior middle temporal cortex when semantic association strength is manipulated in the context of semantic similarity judgments (Wagner et al., 2001), or lexico-semantic categorization (Roskies et al., 2001). Specifically, increased activation in dorsal and ventral prefrontal cortices is associated with weak rather than strong semantic association. A recent fMRI study (Jackson et al., 2015) also reported graded activation differences associated with levels of semantic task difficulty. An example is the increased activation in the inferior frontal regions for the conceptual similarity judgment—the condition associated with the longest response time—compared to the associative similarity condition. Taken together, these results support the idea that the processing of conceptual semantic similarity activates the inferior frontal regions, as it requires more effortful semantic processing.

In summary, it appears that the task-related differences in activation reported here by reference to three levels of semantic relationships (functional, typical, and atypical) does not reflect the difference in semantic relationship type itself, but rather increasing levels of difficulty of achieving the word-matching task. Indeed, the inter-semantic rules comparison performed in the present study shows relatively similar neurofunctional networks for all types of semantic relationship (Figures 4A,B; Tables 7, 8) regardless of age, including the prefrontal, the inferior parietal and the temporal areas (anterior and posterior parts), all of them specifically involved in semantic control. This proposal is largely consistent with the results reported in a study conducted by Jackson et al. (2015).

Furthermore, the second main finding relates to a pattern of activation that occurs when the older adults were compared to the younger ones (Tables 4–6). Our results show that some prefrontal regions are stronger activated in older participants in order to allow for good performance, even at the less demanding level of semantic control demand (Figure 3). This suggests that in order to cope with the task demand, the older adults recruit more executive neural resources as compared to the younger. This activation pattern suggests that the level of the strength of the association between the paired words impacts younger and older adults differently leading to additional cognitive control resources allocation by older participants. This result is compatible with what has been observed, and was predicted by reference to the CRUNCH model. Indeed, the older adults here engaged in the repetition of the application of all three semantic rules exhibit activations in the inferior prefrontal areas (frontopolar, orbitofrontal, and ventrolateral PFC). Considering that our older adults group are very highly educated (an average of 17 years), the age-related brain activation changes are consistent with neuroimaging studies indicating that neurofunctional reorganization phenomenon tends to be observed at a cognitive level that is present mainly in well-educated individuals (Springer et al., 2005). Along these lines, some language studies have reported that greater fluency performance in older adults, relative to younger ones, might have been related to higher levels of education using broader vocabulary, underpinned by efficient strategies (e.g., Bolla et al., 1990; Tombaugh, 1999; Kahlaoui et al., 2012). Other evidence resulting from the cognitive reserve concept (Stern, 2002, 2009) suggests a contribution from verbal proxies, owing to subjects having undergone years of formal education, of an efficient network selection which could take advantage of extensive neural resources. Similarly, Barulli and Stern (2013) have also reported that a higher cognitive reserve based on a high verbal intelligence quotient and years of education in healthy, older, adults reflects an ability to better neural resources allocation in successfully performing verbal tasks. On the basis of evidence showing knowledge-driven expertise through a life span, preservation of semantic processing could be associated with adaptive and unique neurofunctional patterns during healthy aging (Cabeza et al., 2002; Aine et al., 2006; Greenwood, 2007; Greenwood and Parasuraman, 2010; Lacombe et al., 2015). However, we believe that more studies are needed to clarify our understanding of the age-related executive processes in semantic tasks according to the level of education. The impact of level of education on the nature and extent of neurofunctional reorganization, according to different level of education (low vs. high) would be performed in the future aging studies.

In addition, we showed that, as semantic control demand increased across semantic relationship type (from functional to typical), the older adults exhibit activation of the inferior prefrontal regions at a greater extent than the younger in order to be able to cope with the increasing task-demands. Furthermore, the older adults were characterized by not only more frontal activation (left DLPFC and the right dorsal medial PFC) but also by activations of the posterior regions, including the left inferior parietal cortex (angular and supramarginal gyri) and the right occipital cortex. Considering a limited resource model in aging, a total amount of available processing resources is not what declines with age, but instead, the efficiency of the engaged neural resources. From a cognitive aging standpoint, some studies have even suggested that there is no age-related executive decline (Boone et al., 1990) and successful aging has been related to flexible and adaptive brain resources (Kramer et al., 1999; Adrover-Roig and Barceló, 2010). These neurofunctional changes could be either the expression of adaptive neurofunctional patterns, or a possible evolution with age of the neurofunctional bases of semantic processing—which would increase emphasis upon the areas expressing differences in the strategies older adults used to resolve the task.

Beyond the activation of ventrolateral prefrontal, frontopolar, and orbitofrontal areas, older adults also recruit the dorsal-medial prefrontal and lateral premotor areas to a greater extent, and bilaterally, in order to support increase task demands. Although, more posterior regions (inferior parietal and occipital regions) are involved in the repetition of the application of the typical rule, older adults recruit additional prefrontal regions (dorso-medial cortex and lateral premotor), and these bilaterally when they are required to apply the atypical rule. There is also evidence that the atypical rule also contains an inhibitory control component, as the subjects have to suppress the dominant response to choose the item that is most closely related (Grossman et al., 2002; Noonan et al., 2013). Indeed, when typicality is low, a word-matching task requires more control of typicality processing to perform. The less typical the item the more taxing it would be to augment less salient semantic features suggesting high selection/inhibitory demands (Jefferies, 2013; Santi et al., 2016). Furthermore, the involvement of more extensive frontal activation in older adults could tend to express the requirement of more inhibitory control when typicality is low during word-matching.

In other words, older adults encounter their limited cognitive resources by recruiting extra prefrontal areas bilaterally. However, this adaptive neurofunctional reorganization appears to have its limits since it is observed that older adults with relatively healthy cognitive abilities appear to reach a critical threshold (CRUNCH phenomenon), after which there is a larger benefit of preserved semantic systems, under effortful semantic control conditions. These results are in accordance with the view that older adults appear to reach their capacity with increasing task demands probably because they become overwhelmed and then cease to effectively perform (Steffener et al., 2014).

Taken together, as more semantic control is required, these results point to a possible specificity of the neurofunctional basis of the complex relationship between the semantics of a word, and its relationship with the executive system. Indeed, whereas in most cognitive domains there seems to be a point at which a continuous increase in task demand reaches a saturation point, the increase in task demand for the semantic pairing of words appears to benefit from a shift from a more semantic control network (left VLPFC, left inferior parietal, occipital) to a more general, less specific executive control (e.g., dorso-medio-prefrontal bilaterally). This wider range of alternative possibilities could serve to explain the relative preservation of word semantic processing in aging.

As was suggested earlier, the current study reports age-related activation changes in the inferior prefrontal regions and the inferior parietal regions, associated with increased semantic control demands, which are consistent with previous studies (Roskies et al., 2001; Noonan et al., 2010). This neurofunctional network is part of a cognitive control network that is engaged when the task requires the participant to face a cognitive processing challenge (Kennedy et al., 2015). This capacity of the aging brain to mobilize the frontal and parietal regions necessary for highly demanding cognitive processes, such as increased semantic control demands, could represent the contribution of neurofunctional resources ranging from specific brain regions involved in maintaining semantic selection and controlled retrieval (inferior PFC, inferior parietal, anterior cingulate; e.g., Binder et al., 2009; Noonan et al., 2013; Peelle et al., 2013), to more widespread recruitment of general-executive control brain areas (dorso-medial PFC and ventrolateral PFC bilaterally), the latter phenomenon having been shown to be consistently activated in cases of executive demands. As we age, neural resource engagement would shift from specific to more general neurofunctional networks, probably because the components of the network which are part of the more canonical semantic control networks have been involved to their full capacity. In line with this suggestion, Roskies et al. (2001) reported that activation of inferior prefrontal areas as modality-specific control regions might be subserving semantic decisions such as determining whether a certain criterion has been met. Thus, greater frontal contribution is reported when semantic relationship between words is ambiguous and requires the reactivation of semantic representations, or the selection of more attributes, thus putting more demands on the cognitive control system.

Intriguingly, in one study (Martins et al., 2014), the inferior prefrontal activations have not been considered as related to task-demands. These authors report neurofunctional changes in older adults when they match words after positive feedback. Dorsolateral prefrontal activation is reported when older participants perform both semantic and phonological rule matching. This similar pattern of neural activation is interpreted as a decrease in neurofunctional specificity with age. It should be noted that the semantic control manipulation demanded in the word-matching task reported here is greater than the demands of the Wisconsin Word Sorting Task used by Martins et al. (2014) to explore semantic and phonological processes. However, absence of brain activation differences concluded by these authors, when comparing different matching rules in the older group, could be explained neither by postulating decreased neurofunctional specificity, nor by distinct levels of their task demands. Therefore, in the present study, age-related neurofunctional reorganization is thought to underlie an ability of older adults to dynamically adapt neurofunctional resources to cope with task-demands.

In human cognition, language repetition helps to temporary maintain an information until a response is produced. As one of the most important concomitant factors to language rehabilitation, repetition promotes not only the learning or relearning of behaviors but also the maintenance of skills over time. The last decades of neuroplasticity research highlights the mechanisms that help create the appropriate and functional neural patterns to improve or restore a lost function (Kleim and Jones, 2008). Among these factors, rehearsal of a new learned or re-learned behavior triggers an adaptive neurofunctional reorganization in the healthy brain as well as after brain damage. More importantly, the results of the present study are in line with the integrative framework proposed by Majerus (2013) that put forth a valuable contribution of short-term maintenance and repetition of verbal information. While studying functional activation gives limited interpretation of how these functional regions are interconnected, the present findings could prospectively aid to further extend previous interpretations of short-term maintenance and repetition of semantic information during language processing.

Three findings of our study highlight this interpretation. Firstly, both language and verbal STM networks are involved during semantic rule repetition regardless of age. Importantly, active maintenance of “complex” semantic representations (atypical semantic relationships) during rule repetition involves sub-cortical regions that support increasing load-effects in verbal STM. These findings largely support the notion of two distinct neural pathways for the maintenance and updating of information (Ekman et al., 2016). Secondly, a high task-demand impact differentially according to age on verbal STM network. More precisely, rather than in language networks, these differences are found in the neural patterns associated with domain-general executive control, including the left superior parietal cortex and the dorso-medial PFC (BA 8) bilaterally. Indeed, to ensure effective use of rules and also its active maintenance in STM, older adults recruit extensively the dorso-medial PFC to support this increase in the complexity of the semantic rule. This leads to a greater demand on STM. This assumption is coherent with the work of Fiebach et al. (2006) on short-maintenance of semantic information. The engagement of this functional brain region was reported in the monitoring of effort during ongoing processing required to keep active rule maintenance in STM in order to achieve semantic matching. Our results are in line with Fiebach's et al. (2006) hypothesis of a frontally-guided activation of temporal semantic representations. These age-related neuro-functional changes within integrative networks help to sustain the notions of variability and dynamic systems as contributing to an understanding of cognitive and neural mechanisms that underlie adaptive changes in healthy cognitive aging.

Thirdly, during rule repetition, the ability of older adults to simply maintain a given rule in their verbal STM has also been reported to be preserved in previous studies (Martins et al., 2014). In the same vein, Kurth et al. (2016) reported increased activation in the dorsal PFC and inferior parietal sulcus (known as part of cognitive control network) with increased verbal STM load. More interestingly, their main findings argue against the notion that aging effects are supported by top- down process engagement. Although, older adults showed less accurate and slower response time as activation increased in the dorsal fronto-parietal cortex, their performance compared to their younger counterparts suggests unimpaired ability to recruit top-down processes to face higher level-loads in verbal STM. Moreover, and consistent with our results, inferior frontal and parietal regions engagement in healthy aging seem to reflect that the cognitive control system is still dynamic and helpful during language comprehension tasks. As suggested by Fedorenko and Thompson-Schill (2014), cognitive control is sometimes necessary and useful for successful language comprehension. According to this view, cognitive control resources would be implicated in preventing language loss in healthy aging (Wingfield and Grossman, 2006; Hoyau et al., 2017). In sum, in the context of learning and rehabilitation, effortful cognitive processing may involve greater executive resources allocation, thus shedding light on the flexible and dynamic way in which cognitive control and language systems interact under high-level semantic processing.

## Conclusion

In conclusion, the present study suggests that the age-related changes in the activation patterns associated with the repetitive application of a semantic rule in a word-matching task can be best accounted for by differences in the semantic control demands between semantic rules. The activation in the inferior prefrontal regions involved during the repetition of the application of a given semantic rule suggests that age-related activation changes in PFC could be observed, even if behavioral performance is maintained. These results are likely to reflect a flexible executive control system that allocates resources across specific cortical regions depending on the demands of semantic processing in language comprehension. Our findings are consistent with the view that neural patterns related to executive control processes support semantic performance in categorization tasks (Mudar et al., 2015). By reference to the CRUNCH model, high semantic control demands appear to be better supported by regions underlying domain-general aspects of cognition rather than language-specific processes. In this respect, our findings are consistent with semantic tasks studied for the high demands they place on executive control processes (Wagner et al., 2001; Whitney et al., 2009). At the same time, the age-related difference between associative and conceptual semantic similarity appears to be related to the extent of semantic control demand rather than to nature of the semantic relationship type. In fact, the differences in activation between the different types of semantic relations used in the present study are also consistent with the hypothesis that this distinction lies in the dynamic coordination of different activation patterns rather than being related to specific brain regions (Mirman et al., 2017). It is clear how the simple repetition of the application of a semantic word-matching rule reveals much about the function of the aging brain and its ability to categorize the world, semantically, through its words.

## Author contributions

Study conception and design: IM, YJ, OM, MW, and J-SP. Acquisition of Data: IM and YJ. Analysis and interpretation of data: IM, YJ, and BP. Drafting of manuscript: IM, YJ, BP, and MA. Critical revision: IM, YJ, BP and MA.

### Conflict of interest statement

The authors declare that the research was conducted in the absence of any commercial or financial relationships that could be construed as a potential conflict of interest.

## Footnotes

^1^www.fmrib.ox.ac.uk/fsl
